# The burden of COPD mortality due to ambient air pollution in Guangzhou, China

**DOI:** 10.1038/srep25900

**Published:** 2016-05-19

**Authors:** Li Li, Jun Yang, Yun-Feng Song, Ping-Yan Chen, Chun-Quan Ou

**Affiliations:** 1 State Key Laboratory of Organ Failure Research, Department of Biostatistics, Guangdong Provincial Key Laboratory of Tropical Disease Research, School of Public Health, Southern Medical University, Guangzhou 510515, China; 2 WHO Collaborating Centre for Infectious Disease Epidemiology and Control, School of Public Health, Li Ka Shing Faculty of Medicine, The University of Hong Kong, Hong Kong Special Administrative Region, China; 3 State Key Laboratory of Infectious Disease Prevention and Control, Collaborative Innovation Centre for Diagnosis and Treatment of Infectious Diseases, National Institute for Communicable Disease Control and Prevention, Chinese Centre for Disease Control and Prevention, Beijing 102206, China; 4 Intensive Care Unit, Guangdong No. 2 Provincial People’s Hospital, Guangzhou 510317, China

## Abstract

Few studies have investigated the chronic obstructive pulmonary disease (COPD) mortality fraction attributable to air pollution and modification by individual characteristics of air pollution effects. We applied distributed lag non-linear models to assess the associations between air pollution and COPD mortality in 2007–2011 in Guangzhou, China, and the total COPD mortality fraction attributable to air pollution was calculated as well. We found that an increase of 10 μg/m^3^ in particulate matter with an aerodynamic diameter of 10 μm or less (PM_10_), sulfur dioxide (SO_2_) and nitrogen dioxide (NO_2_) was associated with a 1.58% (95% confidence interval (CI): 0.12–3.06%), 3.45% (95% CI: 1.30–5.66%) and 2.35% (95% CI: 0.42–4.32%) increase of COPD mortality over a lag of 0–15 days, respectively. Greater air pollution effects were observed in the elderly, males and residents with low educational attainment. The results showed 10.91% (95% CI: 1.02–9.58%), 12.71% (95% CI: 5.03–19.85%) and 13.38% (95% CI: 2.67–22.84%) COPD mortality was attributable to current PM_10_, SO_2_ and NO_2_ exposure, respectively. In conclusion, the associations between air pollution and COPD mortality differed by individual characteristics. There were remarkable COPD mortality burdens attributable to air pollution in Guangzhou.

Worldwide, chronic obstructive pulmonary disease (COPD) affects 329 million people^1^. COPD has become the third-leading cause of death^2^, resulting in 2.9 million deaths globally in 2013^3^. The number is expected to rise due to an increasing number of smokers and an aging population in many countries^4^.

An Official Public Policy Statement by the American Thoracic Society indicates that the exposure to ambient air pollution, in public health terms, likely contributes tremendously to the growing global burden of COPD^5^. Regarding the relationships between air pollution and COPD mortality, Zanobetti *et al*.^6^ found that particles were correlated with mortality of COPD patients in 34 US cities in 1985–1999, with a hazard ratio of 1.22 (95% confidence interval (CI): 1.17–1.27) for an increase of 10 μg/m^3^ in particulate matter with an aerodynamic diameter of 10 μm or less (PM_10_). A longitudinal study^7^ investigated the associations of long-term exposure to elevated traffic-related air pollution with the risk of COPD mortality in metropolitan Vancouver, Canada, and exposure-response trends for these associations were observed. A few time-series studies^8^,^9^,^10^,^11^,^12^ analyzed the short-term effects of air pollution on COPD mortality, which commonly assessed the effects within a week; however, our prior study^13^ indicated that the associations between the Air Pollution Index (API) and mortality lasted for 15 days. Accordingly, examining the relationships between specific air pollutants and COPD mortality within several days may not reveal the complete picture of the associations. Broader time windows are required to explore the time courses of the associations. Few studies assessed the potential modification of individual characteristics on the short-term effects of air pollution on COPD mortality. Further investigations are needed to strengthen our understanding of ambient air pollution-associated COPD mortality.

Regarding the associations between air pollution and COPD mortality, most previous studies used ratio measures, such as relative risk (RR) and odds ratio (OR), in the analysis. Compared with these indices, the attributable deaths (AD) and attributable fraction (AF) provide more information on the excess burden due to air pollution exposure, which are more suitable for estimating potential benefits from interventions aimed at reducing air pollution. AF, compared with RR and OR, has more public health consideration because it directly provides the proportion of people who may die due to air pollution exposure among all deaths. Norman *et al*.^14^ calculated the COPD deaths attributable to urban particulate matters in South Africa in 2000. Compared to AD, AF was not associated with population size or the total number of deaths and therefore can be used to compare the results from different regions. The estimate of AF due to the exposure to ambient air pollutants, including gaseous pollutants, can provide more comprehensive information on the COPD mortality burden of air pollution.

More than 90% of global COPD deaths occur in low- and middle-income countries^15^. In China, rapid demographic changes in the past few decades, such as an increasing number of Chinese living in the COPD age range, make COPD an inevitable public health challenge in the foreseeable future. Guangzhou is the largest city in southern China where the burden of COPD is heavy, with 8 persons dying due to COPD per day in 2007–2008^8^. Meng *et al*.^8^ provided the RR estimates for the association between air pollution and mortality using two-year data (2007–2008). The present study further assessed the burden of COPD mortality due to exposure to ambient air pollution using AF and examined the potential effect modification of air pollution by individual characteristics in Guangzhou, China, in 2007–2011.

## Results

There were 10,095 COPD deaths in 2007–2011 in Guangzhou, accounting for 8.4% of all registered deaths. 5.2% of them were <65 years of age. 60.1% were males, and 73.2% were residents with low educational attainment (Table 1).

Over the 1,826 days of the study, an average of 6 COPD deaths (range: 0 to 22) occurred per day, and the mean daily concentration of PM_10_, SO_2_ and NO_2_ was 72.6 μg/m^3^, 40.0 μg/m^3^ and 60.7 μg/m^3^, respectively (Table 2). Of the total days, 4.76%, 8.05% and 1.10% of days did not achieve the air quality target of the Chinese Ministry of Environmental Protection 2012 (150 μg/m^3^, 80 μg/m^3^ and 150 μg/m^3^, respectively), and 67.9% and 73.9% of days did not achieve the target of WHO Air Quality Guidelines for PM_10_ and SO_2_ (50 μg/m^3^ and 20 μg/m^3^, respectively).

**Figure 1:**
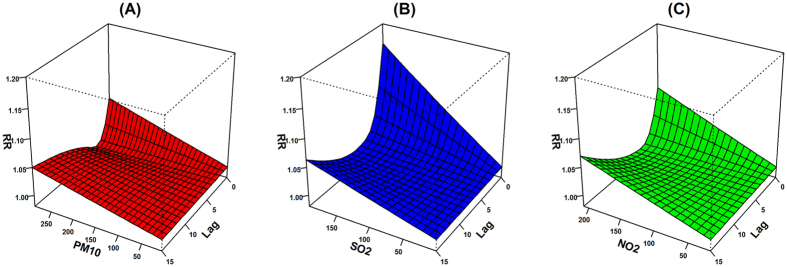
3-D plot of percentage change in COPD mortality associated with 10 μg/m^3^ increases in air pollutants along ambient air pollutant concentrations and lag days.

**Figure 2:**
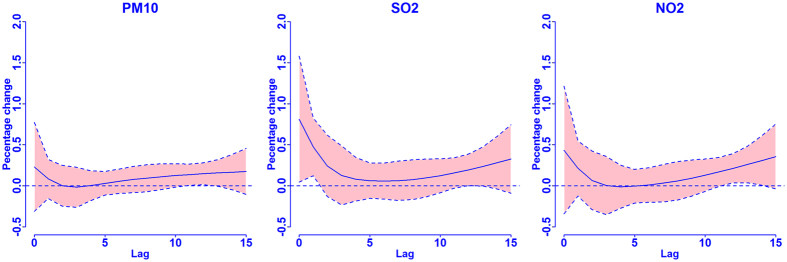
The percentage change in COPD mortality associated with 10 μg/m^3^ increases in ambient air pollutant concentrations along lag 0–15 days.

The distributed lag surface reveals that COPD mortality risk was positively associated with air pollutant concentrations, with the strongest single-day association on the current day for all three air pollutants (Fig. 1). The harvesting effects were observed for PM_10_ and NO_2_ on lag 2–7 days (Fig. 2). An increase of 10 μg/m^3^ in the concentration of PM_10_, SO_2_ and NO_2_ was associated with a cumulative 1.58% (95% CI: 0.12–3.06%), 3.45% (95% CI: 1.30–5.66%) and 2.35% (95% CI: 0.42–4.32%) increase, respectively, in COPD mortality across lag 0–15 days (Table 3). The effect estimates for the current day’s air pollutant concentrations are provided in the Supplementary Table 2.

Greater PM_10_, SO_2_ and NO_2_ effects were observed for the elderly and males. Additionally, residents with low educational attainment were more susceptible to the air pollution effects (Table 3).

Based on the estimates of RR, we calculated that 10.91% (95% CI: 1.02–19.58%), 12.71% (95% CI: 5.03–19.85%) and 13.38% (95% CI: 2.67–22.84%) COPD mortality were attributable to current PM_10_, SO_2_ and NO_2_ exposures, respectively. If the concentrations of PM_10_, SO_2_ and NO_2_ attained the levels of Chinese national ambient air quality standards (NAAQS), a reduction of 0.23% (95% CI: 0.02–0.44%), 0.66% (95% CI: 0.25–1.07%) and 0.05% (95% CI: 0.01–0.08%) in COPD mortality would be achieved, respectively. If the air pollution decreased to half of the NAAQS levels, the reduction in COPD mortality for PM_10_, SO_2_ and NO_2_ were approximately 10, 5 and 30 times as much as that for the attainment of NAAQS. The corresponding reductions were 4.31% (95% CI: 0.38–7.94%) and 7.15% (95% CI: 2.82–11.24%) if the concentrations of PM_10_ and SO_2_ attained the WHO targets, respectively (Table 4). Because there is no guideline for 24-hour concentrations of NO_2_ recommended by the WHO, the estimate for NO_2_ could not be calculated herein.

Results were robust when we used different models to assess the effects of air pollutants (see Supplementary Table S3).

## Discussion

As a time-series study, this study examined the associations between daily variation in air pollution levels and daily changes in COPD mortality. Those traditional risk factors (e.g., smoking) that were time-independent or did not change on a daily basis were controlled for by design. Our study confirmed that ambient air pollution was associated with COPD mortality in Guangzhou, China. Stronger air pollution effects were observed in the elderly and males. Additionally, residents with low educational attainment were more sensitive to the air pollution effects. The COPD mortality burden attributable to SO_2_ was particularly high (Table 3, Table 4).

Some studies^8^,^9^,^10^,^11^,^12^ examined the associations between air pollution and COPD in different cities. Supplementary Table S4 shows the results of these studies, which indicated that the associations differed by study location. There are several possible reasons for the observed geographical differences. First, different geographical and meteorological patterns of these locations influence the transport and diffusion of air pollutants and chemical reactions between pollutants. Thus, the components of the pollution mixture the residents were exposed to varied by study location. Next, the ability to pay for a suitable medical treatment for COPD may be an alternative explanation for the disparity in the effects of air pollution. COPD patients who could not receive appropriate treatment because of poverty and low health care utilization may be more vulnerable to air pollution effects. Moreover, different parameters used to control for the effects of temperature may also influence the effect estimates of air pollutants. The present study controlled for the potential effects of the current day and previous 14 days’ temperatures because some studies have confirmed that temperature effects were much larger than those of air pollution and lasted for almost two weeks^16^. However, previous studies commonly controlled for the confounding effects of temperature within shorter days, which may overestimate the air pollution effects. Finally, the lag selected for the effects of air pollutants may explain the different effect estimates.

Examining the effect modification of individual characteristics will help identify vulnerable subgroups that are targeted subpopulations for the setting of air quality standards and development of intervention programs. Our study further confirmed that old people were more vulnerable to exposure to air pollutants. Old people, especially those with COPD, should reduce physical exertion, particularly outdoors when the level of air pollutant exceeds the air quality standard.

Although some studies reported stronger air pollution effects among women than among men^17^, the present study showed that males with COPD were more sensitive to air pollution effects. A 20-year prospective California Adventists Health Study^18^ showed similar gender differences in which generally significantly reduced lung function related to air pollution (PM_10_, SO_4_ and ozone) was observed in males, particularly males with parental respiratory illness, but not in females. Possible explanations for gender differences in air pollution effects include confounding or modification effects by smoking behaviors, job-related chemical exposures, and distinct exposure and responses to psychosocial stressors^17^.

We found that COPD patients with low educational attainment were more susceptible to air pollution exposure. This finding is plausible. First, most COPD patients with low educational attainment suffer from financial crisis; thus, they often live in places of low quality that offer little protection from indoor infiltration of air pollution. Additionally, the antioxidant properties of certain nutrients, such as vitamin C, beta carotene, selenium and copper, may modulate an individual’s susceptibility to oxidative damage, and it was suggested that a diet rich in omega-3 fatty acids may inhibit arachidonic acid production, thereby protecting against bronchial constriction^19^. Additionally, a diet rich in fruit and vegetables was found to reduce the risk of COPD^20^. However, COPD patients with low educational attainment commonly have limited access to fresh and nutritious food, resulting in reduced intake of antioxidant polyunsaturated fatty acids and vitamins that may protect against adverse consequences of COPD and particle exposure^21^. Moreover, owing to a lack of relevant education, they may pay less attention to the sanitary conditions of their houses, which exposes them to various risk factors, such as viruses and bacteria. Because increased exposure to viral infections may increase their susceptibility, they tend to have a higher prevalence of diseases that predispose to or can be exacerbated by air pollution. Finally, COPD is a burdensome disease, so COPD patients with low educational attainment commonly receive inferior medical treatment, which means that if they are severely ill, they are more likely to die.

Some biological plausibility has been proposed for the associations between air pollution and COPD mortality. COPD patients commonly have a systemic deficit in their antioxidant defences^22^. The exposure to air pollutants may produce additive oxidative stress as a response to the inflammation of the lungs. Specifically, the exposure to NO_2_ might increase the release of inflammatory mediators from bronchial epithelial cells^23^ and enhance the recruitment of macrophages and T lymphocytes to the airway^24^. In addition, Dadvand *et al*.^25^. showed positive associations between short-term exposures to NO_2_ and C-reactive protein and fibrinogen, with a suggestive association with other inflammatory biomarkers. SO_2_ may produce an immediate irritant effect on the respiratory mucosa because of its high water-solubility in the upper respiratory tract^26^. Moreover, in a rat model, urban air particles induced the expression of genes involved in airway wall fibrosis^27^. Further, air pollution exposure may negatively affect the development of lung function early in life^5^. Additional research is needed to confirm the causal effects of ambient air pollution.

Driscoll *et al*.^28^ assessed the global burden of COPD mortality due to occupational airborne exposures in the year 2000. They found that the AF varied from 9% in some areas of the US with high child and adult mortality to 16% in areas of Europe with low child mortality but high adult mortality and areas of the Western Pacific with very low mortality for both children and adults. Norman *et al*.^29^ showed that 23.2% of COPD mortality in South African children and adults in 2000 was attributable to household use of solid fuels. Norman *et al*.^14^ found that in 2000 there were 466 COPD deaths attributable to urban ambient air pollution (PM_10_ and PM_2.5_) in South Africa. The present study revealed that 10.91%, 12.71% and 13.38% of COPD mortality was attributable to ambient PM_10_, SO_2_ and NO_2_ in Guangzhou, respectively. Notably, we found that more benefits could be achieved from the continuous reduction of air pollution, even if air quality attained Chinese national standards or WHO targets, suggesting that the current standards may not adequately protect people.

Compared to PM_10_ and NO_2_, SO_2_ posed a heavier burden of COPD mortality because of its higher RR and more days with a high SO_2_ level. Although the municipal government introduced regulation to control SO_2_ emissions in 2005, the consumption of coal in Guangzhou is still high, accounting for approximately 70% of total energy consumption. Given the high public health burden of SO_2_ pollution, the government should continue taking some effective measures to control it.

The present study has some limitations. First, we presented only the associations between COPD mortality and three criteria pollutants. Ozone and PM_2.5_ linked to COPD were not considered in this study because the data were not available during the study period. Next, the average air pollutant concentrations collected from seven monitoring stations were used as the average exposure level of air pollution, resulting in measurement error, which may underestimate the air pollution effects. Finally, we considered only the situation in central Guangzhou; no information was provided for rural areas.

In conclusion, ambient air pollution has a significant impact on COPD mortality. Among COPD patients, old people, males and residents with low educational attainment were vulnerable groups in relation to air pollution effects. More attention should be paid to these subpopulations. Our findings indicated that COPD mortality in Guangzhou may decrease by 4.31% and 7.15% if the level of PM_10_ and SO_2_ is reduced to the target values of WHO guidelines, respectively. Public efforts to prevent COPD should include the reduction of ambient air pollution.

## Methods

### Data

Guangzhou is the largest southern city in China. According to the sixth national population census in 2010, there were over 12.7 million permanent residents in Guangzhou, among which 60.8% lived at six urban central districts. We obtained individual data for all registered deaths due to an underlying cause of COPD (the International Classification of Diseases, the tenth version [ICD-10]: J40-J44 and J47) at six urban central districts in Guangzhou between January 1, 2007, and December 31, 2011, from Guangzhou Centre for Disease Control and Prevention. Age group (<65 years and ≥65 years), gender and educational attainment (low educational attainment was defined as illiterate or primary school, high educational attainment as middle school or above) were analyzed as potential effect modifiers for air pollution effects.

The Guangzhou Bureau of Environmental Protection provided daily data for three criteria ambient air pollutants—PM_10_, sulfur dioxide (SO_2_) and nitrogen dioxide (NO_2_)—from seven fixed-site air monitoring stations, with various types of sites located in five different central districts. Detailed descriptions of monitoring stations were described in our previous studies^30^,^31^. The average daily air pollutant concentrations in the entire territory of Guangzhou were computed using the centering method^32^. Daily meteorological data on mean temperature, relative humidity and atmospheric pressure were obtained from the China Meteorological Data Sharing Service System.

### Statistical methods

We used quasi-Poisson regression models combined with distributed lag non-linear models (DLNMs) to analyze the associations between air pollution and COPD mortality. The model is expressed as follows:


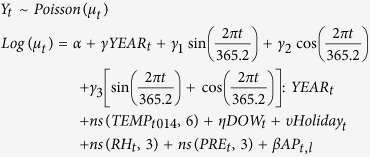


where *t* is the calendar day of observation; *Y*_*t*_ is the observed daily death counts on day *t*, following the quasi-Poisson distribution allowing for over-dispersion. The daily number of COPD deaths was 6, on average. Because only 28 days (1.26%) had a zero count, the zero-inflated Poisson regression was not required. In the present study, we tried different ways to control for the long-term trend, seasonality and effect of temperature. The partial autocorrelation function (PACF), Akaike information criterion (AIC) and generalized cross validation (GCV) were used to select the best model. Details are shown in the Supplementary Material. The PACF for each model was provided in Supplementary Figs S1–S3, and the sum of absolute PACF over 30 days, AIC and GCV for each model is shown in Supplementary Table S1. In the final model, we used dummy variables indicating the year (*YEAR*_*t*_) to control for the long-term trend of COPD mortality. Trigonometric functions were used to control for the seasonality of COPD mortality, and the seasonality was assumed to vary by year. *γ*_*1*_ and *γ*_*2*_ are coefficients for 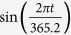 and 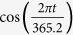, respectively. *γ*_*3*_ is the vector of coefficients for 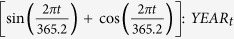. ns(.) is a natural cubic spline. *TEMP*_*t014*_ represents the 15-day moving average temperature on day *t. DOW*_*t*_ are dummy variables indicating the day of the week on day *t*, and *η* is the vector of coefficients. *Holiday*_*t*_ is an indicator variable that is “1” if day *t* was a holiday, and *υ* is the coefficient. A 3 *df* ns was used to smooth the mean relative humidity (*RH*_*t*_) and atmospheric pressure (*PRE*_*t*_). *AP*_*t,l*_ are matrices obtained by applying the DLNM to air pollutant concentrations. We used a linear function for air pollutant concentrations with the maximum lag of 15 days^13^. The *df* for lag was specified to be 4^13^*. β* is the vector of coefficients for *AP*_*t,l*_, and *l* is lag days for air pollutant concentrations.

We plotted 3-D graphics to show the COPD mortality risk along the air pollutant concentrations and lag days. The associations were presented as the percentage change in COPD mortality associated with a 10 μg/m^3^ increase in air pollutant concentrations. Further, we performed subgroup analyses by age group, gender and educational attainment.

To estimate the COPD mortality risk attributable to air pollution, the levels set by the Chinese national ambient air quality standards (NAAQS)^33^ and WHO air quality guidelines^34^ were considered as the references, respectively; that is, the percentage reduction in COPD mortality if the air quality level attained NAAQS or WHO targets. In addition, we also calculated the AFs using 50%, 20% and 0% of the levels set by NAAQS as references. The overall cumulative RR corresponding to each day’s air pollutant concentration was used to compute the AF and AD^35^:


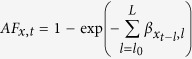



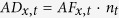


where the *AF*_*x,t*_ and *AD*_*x,t*_ are attributable fraction and attributable deaths at day *t*, respectively; *β*_*x*_ represents the risk associated with the exposure to air pollutants at level *x* (i.e., 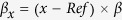; *Ref* is the air quality guideline; 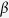 is the coefficient for DLNM of air pollutant concentration). *L* is the maximum lag for the air pollution effects (i.e., 15). *n*_*t*_ is the observed number of COPD deaths at day *t*.

The total AD due to air pollution was given by the sum of the contributions from all the days of the series under study, and its ratio with the total number of observed deaths provided the total AF. Their empirical confidence intervals were obtained by Monte Carlo simulations assuming a multivariate normal distribution of the best linear unbiased predictions of coefficients.

Results of better models judged by the three criteria mentioned above are shown as sensitivity analyses. All analyses were performed using R software (version 3.2.0; R Foundation for Statistical Computing, Vienna, Austria).

## Additional Information

**How to cite this article**: Li, L. *et al*. The burden of COPD mortality due to ambient air pollution in Guangzhou, China. *Sci. Rep.* **6**, 25900; doi: 10.1038/srep25900 (2016).

## Supplementary Material

Supplementary Information

## Figures and Tables

**Table 1:** Demographic characteristics of COPD deaths in Guangzhou, China, 2007–2011.

Factor	Groups	COPD deaths (%)
Age group	<65 years	527 (5.2)
	≥65 years	9 568 (94.8)
Gender	Male	6 063 (60.1)
	Female	4 032 (39.9)
Educational attainment#	Low	7154 (73.2)
	High	2624 (26.8)
Total		10 095 (100.0)

^#^There were 317 subjects for whom information about educational attainments was missing, so we calculated the proportion as the number of each category divided by the sum of two categories.

**Table 2:** Summary statistics for daily number of COPD deaths, daily air pollutant concentrations and weather conditions in Guangzhou, China, 2007–2011.

Variables	Mean ± SD	Minimum	Percentile			Maximum
			25th	50th	75th	
Daily COPD deaths	6 ± 3	0	4	5	7	22
PM_10_ (μg/m^3^)	72.6 ± 37.7	7.6	45.4	65.1	91.1	295.8
SO_2_ (μg/m^3^)	40.0 ± 27.4	2.4	20.0	34.3	52.6	198.5
NO_2_ (μg/m^3^)	60.7 ± 27.0	17.6	41.1	53.9	73.4	213.3
Mean temperature (°C)	22.5 ± 6.4	5.4	18.0	24.0	27.7	33.5
Mean humidity (%)	71.8 ± 13.2	25.0	64.0	73.0	82.0	99.0
Mean pressure (hpa)	10 077 ± 69	9 887	10 026	10 072	10 130	10 266

**Table 3:** The percentage change and 95% CI in mortality from COPD associated with a 10 μg/m^3^ increase in air pollutant concentrations at lag 0–15 days.

Factors	PM_10_	SO_2_	NO_2_
All	1.58 (0.12–3.06)	3.45 (1.30–5.66)	2.35 (0.42–4.32)
Age (years)			
<65	−3.02 (−9.04–3.39)	−1.22 (−9.86–8.25)	−6.52 (−14.12–1.75)
≥65	1.83 (0.32–3.37)	3.77 (1.54–6.06)	2.86 (0.86–4.90)
Gender			
Male	2.04 (0.19–3.93)	4.47 (1.72–7.30)	2.75 (0.30–5.27)
Female	0.91 (−1.42–3.29)	1.93 (−1.47–5.45)	1.76 (−1.31–4.92)
Educational attainment			
Low	2.21 (0.48–3.96)	3.57 (1.04–6.16)	2.90 (0.64–5.22)
High	0.14 (−2.68–3.04)	2.90 (−1.31–7.29)	0.97 (−2.80–4.88)

**Table 4:** The reduction in COPD mortality (%) if air pollution attained the target levels from 2007 to 2011.

Target levels (μg/m^3^)#	Attributable fraction (%, 95% CI)#		
	PM_10_	SO_2_	NO_2_
No pollution (0,0,0)	10.91 (1.02–19.58)	12.71 (5.03–19.85)	13.38 (2.67–22.84)
NAAQS (150,80,150)	0.23 (0.02–0.44)	0.66 (0.25–1.07)	0.05 (0.01–0.08)
50% of NAAQS (75,40,75)	2.27 (0.20–4.21)	3.50 (1.34–5.58)	1.49 (0.28–2.67)
20% of NAAQS (30,16,30)	6.72 (0.60–12.28)	8.13 (3.22–12.74)	7.15 (1.37–12.50)
WHO targets (50,20,-)	4.31 (0.38–7.94)	7.15 (2.82–11.24)	—

^#^The target levels of PM_10_, SO_2_ and NO_2_ are shown in parentheses; NAAQS: Chinese national ambient air quality standards.
