# Supplementary material for: The burden of COPD mortality due to ambient air pollution in Guangzhou, China
Source: Sci Rep. 2016 May 19;6:25900. doi: 10.1038/srep25900 (PMC4872534; doi:10.1038/srep25900)
Supplement: Supplementary Information [file srep25900-s1.pdf]

Supplementary Material

The burden of COPD mortality due to ambient air pollution in Guangzhou, China

Li Li[1,2#], Jun Yang[1,3#], Yun-Feng Song[4], Ping-Yan Chen[1], Chun-Quan Ou[1*]

[1] State Key Laboratory of Organ Failure Research, Department of Biostatistics, Guangdong Provincial Key Laboratory of Tropical Disease Research, School of Public Health and Tropical Medicine, Southern Medical University, Guangzhou 510515, China

[2] WHO Collaborating Centre for Infectious Disease Epidemiology and Control, School of Public Health, Li Ka Shing Faculty of Medicine, The University of Hong Kong, Hong Kong Special Administrative Region, China

[3] State Key Laboratory of Infectious Disease Prevention and Control, Collaborative Innovation Centre for Diagnosis and Treatment of Infectious Diseases, National Institute for Communicable Disease Control and Prevention, Chinese Centre for Disease Control and Prevention, Beijing 102206, China

[4] Intensive Care Unit, Guangdong No.2 Provincial People's Hospital, Guangzhou 510317, China

[#] The authors contributed equally

[*]Corresponding author

We considered the following 16 models in our study:

Model1

$$Y_t \sim Poisson(\mu_t)$$

$$Log(\mu_t) = \alpha + \gamma YEAR_t + \gamma_1 \sin(\frac{2\pi t}{365.2}) + \gamma_2 \cos(\frac{2\pi t}{365.2}) + \gamma_3 \left[\sin(\frac{2\pi t}{365.2}) + \cos(\frac{2\pi t}{365.2}) \right] : YEAR_t$$
$$+ ns(TEMP_{t014}, df = 6) + \eta DOW_t + \upsilon Holiday_t + ns(RH_t, 3) + ns(PRE_t, 3) + \beta AP_{t,l}$$

Model2

$$Y_t \sim Poisson(\mu_t)$$

$$Log(\mu_t) = \alpha + \gamma YEAR_t + \gamma_1 \sin(\frac{2\pi t}{365.2}) + \gamma_2 \cos(\frac{2\pi t}{365.2})$$
$$+ ns(TEMP_{t014}, df = 6) + \eta DOW_t + \upsilon Holiday_t + ns(RH_t, 3) + ns(PRE_t, 3) + \beta AP_{t,l}$$

Model3

$$Y_t \sim Poisson(\mu_t)$$

$$Log(\mu_t) = \alpha + \gamma YEAR_t + \gamma_1 \sin(\frac{2\pi t}{365.2}) + \gamma_2 \cos(\frac{2\pi t}{365.2}) + \gamma_3 \sin(\frac{2\pi t}{365.2/2}) + \gamma_4 \cos(\frac{2\pi t}{365.2/2})$$
$$+ ns(TEMP_{t014}, df = 6) + \eta DOW_t + \upsilon Holiday_t + ns(RH_t, 3) + ns(PRE_t, 3) + \beta AP_{t,l}$$

Model4

$$Y_t \sim Poisson(\mu_t)$$

$$Log(\mu_t) = \alpha + \gamma YEAR_t + \gamma_1 \sin(\frac{2\pi t}{365.2}) + \gamma_2 \cos(\frac{2\pi t}{365.2}) + \gamma_3 \sin(\frac{2\pi t}{365.2/2}) + \gamma_4 \cos(\frac{2\pi t}{365.2/2}) + \gamma_5 \sin(\frac{2\pi t}{365.2/3}) + \gamma_6 \cos(\frac{2\pi t}{365.2/3})$$
$$+ ns(TEMP_{t014}, df = 6) + \eta DOW_t + \upsilon Holiday_t + ns(RH_t, 3) + ns(PRE_t, 3) + \beta AP_{t,l}$$

Model5

$$Y_t \sim Poisson(\mu_t)$$

$$Log(\mu_t) = \alpha + \gamma YEAR_t + \gamma_1 \sin(\frac{2\pi t}{365.2}) + \gamma_2 \cos(\frac{2\pi t}{365.2}) + \gamma_3 \sin(\frac{2\pi t}{365.2/2}) + \gamma_4 \cos(\frac{2\pi t}{365.2/2})$$
$$+ \gamma_5 \sin(\frac{2\pi t}{365.2/3}) + \gamma_6 \cos(\frac{2\pi t}{365.2/3}) + \gamma_7 \sin(\frac{2\pi t}{365.2/4}) + \gamma_8 \cos(\frac{2\pi t}{365.2/4})$$
$$+ ns(TEMP_{t014}, df = 6) + \eta DOW_t + \upsilon Holiday_t + ns(RH_t, 3) + ns(PRE_t, 3) + \beta AP_{t,l}$$

Model6

$$Y_t \sim Poisson(\mu_t)$$

$$Log(\mu_t) = \alpha + \gamma YEAR_t + \gamma_1 \sin(\frac{2\pi t}{365.2}) + \gamma_2 \cos(\frac{2\pi t}{365.2}) + \gamma_3 \sin(\frac{2\pi t}{365.2/2}) + \gamma_4 \cos(\frac{2\pi t}{365.2/2}) + \gamma_5 \sin(\frac{2\pi t}{365.2/3})$$
$$+ \gamma_6 \cos(\frac{2\pi t}{365.2/3}) + \gamma_7 \sin(\frac{2\pi t}{365.2/4}) + \gamma_8 \cos(\frac{2\pi t}{365.2/4}) + \gamma_9 \sin(\frac{2\pi t}{365.2/5}) + \gamma_{10} \cos(\frac{2\pi t}{365.2/5})$$
$$+ ns(TEMP_{t014}, df = 6) + \eta DOW_t + \upsilon Holiday_t + ns(RH_t, 3) + ns(PRE_t, 3) + \beta AP_{t,l}$$

Model7

$$Y_t \sim Poisson(\mu_t)$$

$$Log(\mu_t) = \alpha + \gamma YEAR_t + \gamma_1 \sin(\frac{2\pi t}{365.2}) + \gamma_2 \cos(\frac{2\pi t}{365.2}) + \gamma_3 \sin(\frac{2\pi t}{365.2/2}) + \gamma_4 \cos(\frac{2\pi t}{365.2/2}) + \gamma_5 \sin(\frac{2\pi t}{365.2/3}) + \gamma_6 \cos(\frac{2\pi t}{365.2/3})$$
$$+ \gamma_7 \sin(\frac{2\pi t}{365.2/4}) + \gamma_8 \cos(\frac{2\pi t}{365.2/4}) + \gamma_9 \sin(\frac{2\pi t}{365.2/5}) + \gamma_{10} \cos(\frac{2\pi t}{365.2/5}) + \gamma_{11} \sin(\frac{2\pi t}{365.2/6}) + \gamma_{12} \cos(\frac{2\pi t}{365.2/6})$$
$$+ ns(TEMP_{t014}, df = 6) + \eta DOW_t + \upsilon Holiday_t + ns(RH_t, 3) + ns(PRE_t, 3) + \beta AP_{t,l}$$

Model8

$$Y_t \sim Poisson(\mu_t)$$

$$Log(\mu_t) = \alpha + \gamma YEAR_t + \gamma_1 \sin(\frac{2\pi t}{365.2}) + \gamma_2 \cos(\frac{2\pi t}{365.2}) + \gamma_3 \left[\sin(\frac{2\pi t}{365.2}) + \cos(\frac{2\pi t}{365.2}) \right] : YEAR_t$$
$$+ \lambda Temp_{t,l2} + \eta DOW_t + \upsilon Holiday_t + ns(RH_t, 3) + ns(PRE_t, 3) + \beta AP_{t,l}$$

Where $Temp_{t,l2}$ are matrices obtained by applying the DLNM to mean temperature. We used a ns for mean temperature with the maximum lag of 14 days and a polynomial function for lag. λ is the vector of coefficients for $Temp_{t,l2}$. l_2 is lag day for mean temperature.[1]

Model9

$$Y_t \sim Poisson(\mu_t)$$

$$Log(\mu_t) = \alpha + \gamma YEAR_t + \gamma_1 \sin(\frac{2\pi t}{365.2}) + \gamma_2 \cos(\frac{2\pi t}{365.2})$$
$$+ \lambda Temp_{t,l2} + \eta DOW_t + \upsilon Holiday_t + ns(RH_t, 3) + ns(PRE_t, 3) + \beta AP_{t,l}$$

Model10

$$Y_t \sim Poisson(\mu_t)$$

$$Log(\mu_t) = \alpha + \gamma YEAR_t + \gamma_1 \sin(\frac{2\pi t}{365.2}) + \gamma_2 \cos(\frac{2\pi t}{365.2}) + \gamma_3 \sin(\frac{2\pi t}{365.2/2}) + \gamma_4 \cos(\frac{2\pi t}{365.2/2})$$
$$+ \lambda Temp_{t,l2} + \eta DOW_t + \upsilon Holiday_t + ns(RH_t, 3) + ns(PRE_t, 3) + \beta AP_{t,l}$$

Model11

$$Y_t \sim Poisson(\mu_t)$$

$$Log(\mu_t) = \alpha + \gamma YEAR_t + \gamma_1 \sin(\frac{2\pi t}{365.2}) + \gamma_2 \cos(\frac{2\pi t}{365.2}) + \gamma_3 \sin(\frac{2\pi t}{365.2/2}) + \gamma_4 \cos(\frac{2\pi t}{365.2/2}) + \gamma_5 \sin(\frac{2\pi t}{365.2/3}) + \gamma_6 \cos(\frac{2\pi t}{365.2/3})$$
$$+ \lambda Temp_{t,l2} + \eta DOW_t + \upsilon Holiday_t + ns(RH_t, 3) + ns(PRE_t, 3) + \beta AP_{t,l}$$

Model12

$$Y_t \sim Poisson(\mu_t)$$

$$Log(\mu_t) = \alpha + \gamma YEAR_t + \gamma_1 \sin(\frac{2\pi t}{365.2}) + \gamma_2 \cos(\frac{2\pi t}{365.2}) + \gamma_3 \sin(\frac{2\pi t}{365.2/2}) + \gamma_4 \cos(\frac{2\pi t}{365.2/2})$$
$$+ \gamma_5 \sin(\frac{2\pi t}{365.2/3}) + \gamma_6 \cos(\frac{2\pi t}{365.2/3}) + \gamma_7 \sin(\frac{2\pi t}{365.2/4}) + \gamma_8 \cos(\frac{2\pi t}{365.2/4})$$
$$+ \lambda Temp_{t,l2} + \eta DOW_t + \upsilon Holiday_t + ns(RH_t, 3) + ns(PRE_t, 3) + \beta AP_{t,l}$$

Model13

$$Y_t \sim Poisson(\mu_t)$$

$$Log(\mu_t) = \alpha + \gamma YEAR_t + \gamma_1 \sin(\frac{2\pi t}{365.2}) + \gamma_2 \cos(\frac{2\pi t}{365.2}) + \gamma_3 \sin(\frac{2\pi t}{365.2/2}) + \gamma_4 \cos(\frac{2\pi t}{365.2/2}) + \gamma_5 \sin(\frac{2\pi t}{365.2/3})$$
$$+ \gamma_6 \cos(\frac{2\pi t}{365.2/3}) + \gamma_7 \sin(\frac{2\pi t}{365.2/4}) + \gamma_8 \cos(\frac{2\pi t}{365.2/4}) + \gamma_9 \sin(\frac{2\pi t}{365.2/5}) + \gamma_{10} \cos(\frac{2\pi t}{365.2/5})$$
$$+ \lambda Temp_{t,l2} + \eta DOW_t + \upsilon Holiday_t + ns(RH_t, 3) + ns(PRE_t, 3) + \beta AP_{t,l}$$

Model14

$$Y_t \sim Poisson(\mu_t)$$

$$Log(\mu_t) = \alpha + \gamma YEAR_t + \gamma_1 \sin(\frac{2\pi t}{365.2}) + \gamma_2 \cos(\frac{2\pi t}{365.2}) + \gamma_3 \sin(\frac{2\pi t}{365.2/2}) + \gamma_4 \cos(\frac{2\pi t}{365.2/2}) + \gamma_5 \sin(\frac{2\pi t}{365.2/3}) + \gamma_6 \cos(\frac{2\pi t}{365.2/3})$$
$$+ \gamma_7 \sin(\frac{2\pi t}{365.2/4}) + \gamma_8 \cos(\frac{2\pi t}{365.2/4}) + \gamma_9 \sin(\frac{2\pi t}{365.2/5}) + \gamma_{10} \cos(\frac{2\pi t}{365.2/5}) + \gamma_{11} \sin(\frac{2\pi t}{365.2/6}) + \gamma_{12} \cos(\frac{2\pi t}{365.2/6})$$
$$+ \lambda Temp_{t,l2} + \eta DOW_t + \upsilon Holiday_t + ns(RH_t, 3) + ns(PRE_t, 3) + \beta AP_{t,l}$$

Model15

$$Y_t \sim Poisson(\mu_t)$$

$$Log(\mu_t) = \alpha + ns(t, 4 \times 5) + +ns(Temp_{t014}, 6) + \eta DOW_t + \upsilon Holiday_t + ns(RH_t, 3) + ns(PRE_t, 3) + \beta AP_{t,l}$$

Model16

$$Y_t \sim Poisson(\mu_t)$$

$$Log(\mu_t) = \alpha + ns(Time_t, 4 \times 5) + \lambda Temp_{t,l2} + \eta DOW_t + \upsilon Holiday_t + ns(RH_t, 3) + ns(PRE_t, 3) + \beta AP_{t,l}$$

Supplementary Table S1 Sum of absolute partial autocorrelation function (PACF), Akaike Information Criterion (AIC) and generalized cross validation (GCV) for each model

Air pollutant	Model	AIC	Sum of absolute PACF	GCV
PM_{10}	Model1	6945.253	0.623	1.099
	Model2	6981.865	0.736	1.119
	Model3	6984.459	0.717	1.121
	Model4	6988.592	0.718	1.124
	Model5	6992.637	0.722	1.127
	Model6	6993.900	0.734	1.128
	Model7	6996.315	0.732	1.130
	Model8	6948.963	0.614	1.102
	Model9	6981.744	0.726	1.120
	Model10	6982.557	0.704	1.121
	Model11	6986.060	0.705	1.123
	Model12	6989.795	0.709	1.126
	Model13	6990.204	0.720	1.126
	Model14	6992.629	0.719	1.128
	Model15	6935.746	0.741	1.093
	Model16	6939.393	0.708	1.097
SO_2	Model1	6938.130	0.599	1.094
	Model2	6971.934	0.681	1.113
	Model3	6972.379	0.644	1.113

	Model4	6976.512		0.644	1.116
	Model5	6980.403		0.649	1.119
	Model6	6981.312		0.665	1.120
	Model7	6983.934		0.666	1.122
	Model8	6941.551		0.594	1.097
	Model9	6970.723		0.671	1.113
	Model10	6967.086		0.628	1.111
	Model11	6970.911		0.627	1.113
	Model12	6974.478		0.632	1.116
	Model13	6974.396		0.644	1.116
	Model14	6977.350		0.643	1.118
	Model15	6930.792		0.733	1.090
	Model16	6932.879		0.704	1.092
NO_2	Model1	6942.983		0.614	1.097
	Model2	6973.085		0.684	1.113
	Model3	6975.925		0.673	1.116
	Model4	6979.999		0.674	1.118
	Model5	6983.845		0.678	1.121
	Model6	6984.778		0.693	1.122
	Model7	6987.192		0.691	1.124
	Model8	6944.939		0.605	1.099
	Model9	6969.191		0.661	1.112

Model10	6970.347		0.641	1.113
Model11	6973.702		0.639	1.115
Model12	6977.128		0.642	1.118
Model13	6976.793		0.656	1.118
Model14	6979.526		0.656	1.120
Model15	6934.721		0.731	1.093
Model16	6937.083		0.692	1.095

Supplementary Table S2 The percentage change and 95% CI in mortality from COPD associated with a 10 μg/m^3 increase in air pollutant concentrations on the current day

Factors	PM_{10}	SO_2	NO_2
All	0.23 (-0.31–0.77)	0.81 (0.05–1.58)	0.43 (-0.34–1.22)
Age (years)			
<65	-0.55 (-2.94–1.90)	0.72 (-2.58–4.13)	-3.03 (-6.43–0.49)
⩾65	0.27 (-0.29–0.83)	0.83 (0.04–1.62)	0.62 (-0.18–1.42)
Gender			
Male	-0.16 (-0.84–0.53)	0.29 (-0.69–1.27)	-0.1 (-1.08–0.90)
Female	0.79 (-0.08–1.66)	1.57 (0.35–2.80)	1.21 (-0.03–2.47)
Educational attainment			
Low	0.41 (-0.23–1.05)	0.93 (0.03–1.84)	0.70 (-0.21–1.62)
High	-0.17 (-1.23–0.90)	0.43 (-1.08–1.96)	-0.35 (-1.88–1.20)

Supplementary Table S3 Results of better models based on three criteria

Air pollutant	Model	Percentage change	AF (China)	AF (WHO)
PM_{10}	Model1	1.58 (0.12–3.06)	0.23 (0.02–0.44)	4.31 (0.38–7.94)
	Model8	1.59 (0.02–3.19)	0.24 (0.01–0.46)	4.34 (0.02–8.43)
	Model15	1.63 (0.07–3.22)	0.24 (0.01–0.45)	4.44 (0.21–8.25)
	Model16	1.91 (0.22–3.63)	0.28 (0.03–0.52)	5.16 (0.53–9.38)
SO_2	Model1	3.45 (1.30–5.66)	0.66 (0.25–1.07)	7.15 (2.82–11.24)
	Model8	3.82 (1.54–6.15)	0.73 (0.30–1.15)	7.85 (3.30–12.21)
	Model15	2.89 (0.57–5.27)	0.55 (0.10–0.99)	6.03 (1.20–10.41)
	Model16	3.80 (1.31–6.35)	0.73 (0.25–1.20)	7.81 (2.83–12.31)
NO_2	Model1	2.35 (0.42–4.32)	0.05 (0.01–0.08)	
	Model8	2.74 (0.59–4.94)	0.06 (0.02–0.10)	
	Model15	2.02 (0.02–4.07)	0.04 (0.00–0.08)	
	Model16	2.55 (0.33–4.81)	0.05 (0.01–0.09)	

[#] AF: attributable fraction.

Supplementary Table S4 Comparison of the impacts of ambient air pollution on COPD mortality (showed as the percentage change per 10 μg/m^3 increase in air pollutant concentrations) in different populations ordered by publication year

**Authors**	**Study location**	**Lag**	**Age group**	**PM_{10}**	**SO_2**	**NO_2**
**Meng et al.**[2]	Beijing	0–1	All–age	0.28 (-0.10–0.67)	0.48 (-0.47–1.43)	0.74 (-0.83–2.31)
	Shanghai	0–1	All–age	0.42 (0.16–0.68)	1.81 (1.06–2.57)	1.79 (1.11–2.48)
	Guangzhou	0–1	All–age	2.10 (1.41–2.79)	1.74 (0.88–2.61)	2.63 (1.78–3.48)
	Hong Kong	0–1	All–age	0.47 (-0.34–1.29)	0.74 (-0.90–2.39)	1.36 (0.40–2.33)
	Meta–analyses	0–1	All–age	0.78 (0.13–1.42)	1.38 (0.92–1.85)	1.85 (1.40–2.29)
**Romieu et al.**[3]	Sao Paulo	0–3	All–age	1.43 (0.69–2.18)		
		0–3	≥65 years	1.81 (0.97–2.65)		
	Rio de Janeiro	0–3	All–age	1.64 (0.19–3.12)		
		0–3	≥65 years	2.58 (1.00–4.19)		
	Porto Alegre	0–3	All–age	4.31 (1.81–6.87)		
		0–3	≥65 years	2.73 (0.00–5.54)		
	Santiago	0–3	All–age	0.82 (0.15–1.50)		
		0–3	≥65 years	1.00 (0.27–1.74)		
	Concepcion	0–3	All–age	3.89 (1.12–6.75)		
		0–3	≥65 years	7.76 (4.80–10.80)		
	Temuco	0–3	All–age	8.37 (6.22–10.56)		
		0–3	≥65 years	6.30 (4.13–8.53)		
	Mexico City	0–3	All–age	1.76 (0.54–3.00)		

		0–3	≥65 years	0.28 (–0.42–0.99)		
	Monterrey	0–3	All–age	2.49 (1.66–3.33)		
		0–3	≥65 years	–0.80 (–1.69–0.10)		
	Toluca	0–3	All–age	–0.03 (–1.65–1.63)		
		0–3	≥65 years	–0.16 (–1.91–1.62)		
	Meta–analyses	0–3	All–age	2.44 (1.36–3.59)		
**Zeka et al.**[4]	20 cities in US	0	All–age	–0.26 (–0.89–0.37)		
		1	All–age	0.43 (–0.24–1.10)		
		2	All–age	0.23 (–0.38–0.84)		
**Fischer et al.**[5]	The Netherlands[#]	0–6	<45 years	15.30 (-41.30–126.80)	-26.30 (-51.0–10.80)	-18.20 (-39.0–9.70)
		0–6	45–64 years	13.90 (-15.90–54.10)	-8.50 (-22.90–8.70)	5.30 (-8.00–20.50)
		0–6	65–74 years	16.60 (-0.90–37.20)	13.70 (3.20–25.10)	17.30 (8.50–26.80)
		0–6	≥75 years	6.60 (-3.50–17.80)	2.60 (-3.30–8.90)	4.60 (0.00–9.40)
		0–3	≥65 years	9.80 (-22.0–223.00)		
**Wong et al.**[6]	Hong Kong	2	All–age		0.00 (–200.00–190.00)	
		0–2	All–age			130.00 (-40.00–310.00)
		0–3	All–age	70.00 (-80.00–230.00)		

[#]For presentation the percentage change was calculated for differences between the 1st and 99th percentile of the air pollution variable. The ranges used were 80 for PM_{10}; 40 for SO_2; 30 for NO_2; all in μg/m^3.

References

1. Li, L. *et al.*, Can the Air Pollution Index be used to communicate the health risks of air pollution? *Environ Pollut* **205** 153 (2015).

2. Meng, X., Wang, C., Cao, D., Wong, C. & Kan, H., Short-term effect of ambient air pollution on COPD mortality in four Chinese cities. *Atmos Environ* **77** 149 (2013).

3. Romieu, I. *et al.*, Multicity study of air pollution and mortality in Latin America (the ESCALA study). *Res Rep Health Eff Inst* 5 (2012).

4. Zeka, A., Zanobetti, A. & Schwartz, J., Short term effects of particulate matter on cause specific mortality: effects of lags and modification by city characteristics. *Occup Environ Med* **62** 718 (2005).

5. Fischer, P., Hoek, G., Brunekreef, B., Verhoeff, A. & van Wijnen, J., Air pollution and mortality in The Netherlands: are the elderly more at risk? *Eur Respir J Suppl* **40** 34s (2003).

6. Wong, T. W., Tam, W. S., Yu, T. S. & Wong, A. H., Associations between daily mortalities from respiratory and cardiovascular diseases and air pollution in Hong Kong, China. *Occup Environ Med* **59** 30 (2002).
